# Meningioma in the third trimester of pregnancy: A case report

**DOI:** 10.1016/j.crwh.2023.e00541

**Published:** 2023-09-09

**Authors:** Carla Ettore, Elisa Zambrotta, Ferdinando Antonio Gulino, Giosuè Giordano Incognito, Francesco Giuseppe Cannone, Giuseppe Ettore

**Affiliations:** Department of Obstetrics and Gynaecology, Azienda di Rilievo Nazionale e di Alta Specializzazione (ARNAS) Garibaldi Nesima, Catania, Italy

**Keywords:** Case reports, Meningioma, Pregnancy

## Abstract

Meningiomas are rare benign tumors during pregnancy. They can put both the mother and the fetus at risk because rapid changes in size may occur. The study reports a case of olfactory groove meningioma in a 34-year-old pregnant woman with visual impairment, anosmia, and dysgeusia that increased in severity during the 29th week of gestation. Magnetic resonance imaging showed an olfactory groove meningioma. The patient underwent a preterm cesarean section to avoid the worsening of the clinical condition due to intracranial compression phenomena. A computed tomography scan of the brain supported the diagnosis. The surgical intervention occurred on the third day after delivery. The clinical course was uneventful and the chiasmal syndrome improved in the following 2 months. Meningiomas in pregnancy may present unique challenges and have a wide array of clinical presentations. Management of maternal meningiomas can be complex and requires a multidisciplinary approach. The treatment decision largely depends on the size and location of the tumor, the presence of symptoms, gestational age, and fetal well-being. Further research is needed to enhance the understanding of the underlying mechanisms and improve management approaches for this rare condition.

## Highlights

• Meningiomas are rare benign tumors during pregnancy.
• Maternal meningiomas can put both the mother and the fetus at risk because rapid changes in size may occur.
• Management of maternal meningiomas can be complex and requires a multidisciplinary approach.
• The treatment decision largely depends on the tumor specifics, symptoms, gestational age, and fetal well-being.

## 1 Introduction

Meningiomas are the most common primary brain tumors, and have a 2:1 female-to-male ratio. They originate from the arachnoid cap cells of the meninges, the protective layers that envelop the brain and spinal cord. They are usually benign and slow-growing but can become problematic due to their location and size [1]. While meningiomas are frequently diagnosed in non-pregnant individuals [1], the presence of a meningioma during pregnancy is rare and can pose unique challenges and considerations for both the mother and the developing fetus [2]. The pathogenesis is still not fully understood, but several factors are thought to play a role [3]. Pregnancy-induced hormonal changes seem to be a major contributory factor and are considered to significantly impact the growth of these tumors [4]. The presence of a meningioma during pregnancy can put both the mother and the fetus at risk because of the rapid changes in meningioma cells and the size of the tumor that may occur. Olfactory groove meningiomas can cause a rapid onset of anosmia and visual loss, accompanied by other symptoms, such as headache, nausea, and vomiting, that might be mistakenly attributed to the pregnancy [5]. The diagnosis typically involves a combination of physical examination, medical history, and imaging studies. Management can be complex and requires a multidisciplinary approach. The treatment decision largely depends on the size and location of the tumor, the presence of symptoms, gestational age, and fetal well-being [6,7].

The present study reports a case of an unrecognized olfactory groove meningioma in a pregnant woman. The report demonstrates how a meningioma can present unique characteristics and how early diagnosis is essential. In this case, pregnancy unmasked the symptoms, necessitating the performance of a preterm cesarean section due to the worsening of the maternal clinical condition.

## 2 Case Presentation

A 34-year-old primigravid woman was admitted to the emergency gynecologic unit reporting anosmia and dysgeusia during the 29th week of pregnancy. She did not report headaches, nausea, or vomiting. Her family and personal history were negative for any significant disease. She affirmed being a former smoker and denied any alcohol or drug abuse.

The symptoms started before pregnancy, and were initially mild. A test at the time for Severe Acute Respiratory Syndrome Coronavirus 2 (SARS-COV-2) was negative and an ophthalmological examination excluded any ocular pathology. Routine first-trimester ultrasound showed no anomalies, and Toxoplasmosis, Other agents, Rubella, Cytomegalovirus, and Herpes simplex (TORCH) screening was negative. During the 22nd week of gestation, she reported blurred vision in both eyes, which became progressively worse, for which she underwent optical coherence tomography. It excluded retina and optic nerve disorders.

On admission, the general physical examination was unremarkable, and the vital signs were within normal limits. A fetal ultrasound scan showed regular fetal growth, homogeneous placenta, regular amniotic fluid, and an umbilical artery Doppler pulsatility index in the 50th centile. The neurological examination did not reveal abnormalities in motor and mental state but her symptoms were an indication for urgent magnetic resonance imaging (MRI) of the brain. It showed a tumor involving the subfrontal middle line starting from the olfactory groove, which marked the anterior part of the corpus callosum, the optic chiasm, and compressed the overlying frontal convolutions, compatible with the diagnosis of an olfactory groove meningioma.

**Fig. 1:**
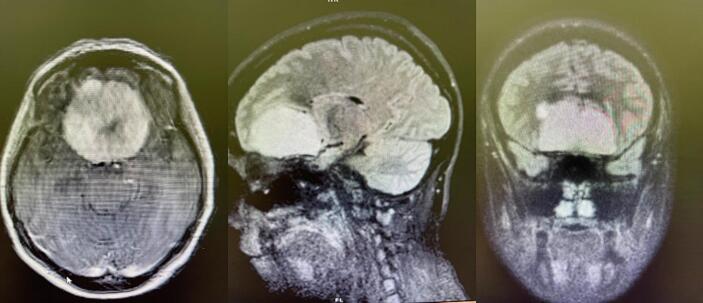
Brain computed tomography with contrast demonstrates a large subfrontal tumor.

After the administration of corticosteroid and magnesium sulfate for fetal lung maturation and neuroprotection, the patient underwent a cesarean section to avoid the worsening of clinical condition due to intracranial compression phenomena. A premature male infant was born, with one- and five-minute Apgar scores of 6 and 8, respectively. A brain computed tomography (CT) scan with contrast demonstrated a homogeneously subfrontal contrast-enhancing mass with well-defined borders and peritumoral brain edema, supporting the earlier diagnosis (Fig. 1).

On the third day after delivery, the patient underwent surgical intervention. The lesion was approached by a frontal craniotomy and complete resection was achieved, with no intraoperative complications. The histopathological examination confirmed the diagnosis. Postoperatively, visual acuity improved, and the visual field returned to normal after 2 months. At the time of writing, the patient had had no relapse and was undergoing follow-up.

## 3 Discussion

Meningiomas are estimated to constitute between 13% and 26% of all intracranial tumors. They show a rising incidence with age and are most common in the sixth and seventh decades of life. In adults, there is a 2:1 female-to-male ratio [1]. The exact prevalence of maternal meningiomas during pregnancy has not been established. However, some epidemiological studies reported that the incidence is low, ranging from 0.1% to 0.5% of all pregnancies. Maternal meningiomas are more common in older pregnant women, possibly due to the age-related increase in the overall incidence of meningiomas [2]. Maternal age at conception, race, and prior radiation exposure are some known risk factors [[3], [4], [5]].

The pathophysiology of meningioma in pregnancy is different from that of other meningiomas and is far from being understood. There are two possible mechanisms to consider: endocrine and vascular. The abundant expression of progesterone receptor (PR) in meningiomas is well established, but it is not known how PR expression is regulated and what function it exercises in tumor growth. It is uncertain if PR contributes to cell proliferation or not; the evidence that several meningiomas enlarge and become symptomatic in pregnancy and reduce their size after delivery suggests an important role of the sex steroid hormone [4]. In a series of 17 gestational meningiomas, Lusis et al. [5] attributed a crucial role in tumor growth to the physiological reversible hemodynamic changes in pregnancy. The increased blood volume and vascularization during pregnancy could also contribute to the rapid growth and symptom manifestation of these tumors. Gestational meningiomas show considerable vascularity, intra- and extracellular edema, and typical foamy, swollen cells, unlike meningiomas in general. The relationship between hormonal and vascular factors needs more clarification. Pregnancy, characterized by significant hormonal fluctuations, may also influence the behavior of preexisting meningiomas, with potential growth or symptom exacerbation. In the present case, mild symptoms began before pregnancy and worsened during the second trimester. Therefore, it is likely that an unrecognized meningioma was already present before pregnancy caused an increase in its size [5].

Maternal meningiomas can present unique challenges during pregnancy. The clinical implications depend largely on the tumor size and location. Common symptoms include headaches, seizures, changes in vision, and neurological deficits like weakness or numbness on one side of the body. The patient complained of anosmia and dysgeusia, but she did not report headache, nausea, or vomiting, providing further evidence of the highly variable symptomatology of this condition.

From the maternal perspective, untreated meningiomas can cause serious neurological complications, and even life-threatening conditions such as hydrocephalus and intracranial hypertension.

From the fetal perspective, maternal meningiomas carry the risk of premature delivery, low birth weight, and, in some severe cases, intrauterine death. Additionally, certain medications for managing maternal symptoms or surgical interventions can have adverse effects on the fetus [5]. In this case, the worsening of clinical symptoms was likely due to a compressive effect with the potential risk of intracranial hypertension.

Diagnosing maternal meningiomas during pregnancy requires a comprehensive evaluation. Imaging studies are crucial in determining the size, location, and characteristics of the tumor. MRI is the preferred imaging technique as it provides a detailed view of the brain and meninges without exposing the mother or fetus to radiation. An early diagnosis can be important for the mother's and fetus's health, because untreated or complex cases can have significant complications, making careful management vital [6].

A multidisciplinary approach was performed involving obstetricians, neonatologists, radiologists, neurosurgeons, and anesthesiologists, which is crucial to manage these cases effectively and ensure the best possible outcomes for both the mother and baby. A “wait and watch” approach with close monitoring might be taken for asymptomatic, small meningiomas [6]. Symptomatic meningiomas often require treatment, which may include medication to manage symptoms like seizures or surgery to remove the tumor [6]. Surgery, including tumor resection or debulking, may be considered if the tumor is causing significant symptoms or poses a risk to the mother or fetus. However, the timing of surgery must be carefully assessed to balance the potential risks to both the mother and the developing baby. Moreover, any surgical intervention must consider the increased risk of complications due to pregnancy-induced physiological changes, such as increased blood volume and coagulation factors. Surgical intervention during pregnancy should be avoided unless demanded by emergency; as far as possible, management should be limited to observation in the first and also second trimester [[7], [8], [9], [10]]. In the present case, the surgical intervention was performed following the preterm cesarean section, carried out to prevent the worsening of maternal complications associated with increased intracranial pressure. The lesion was accessed through a frontal craniotomy, and complete resection was successfully achieved without any intraoperative complications.

The prognosis of maternal meningiomas depends on several factors, including tumor characteristics, treatment modalities, and the overall health of the mother. Most meningiomas are benign, and with timely diagnosis and appropriate management, the prognosis is generally favorable with a low recurrence rate after complete surgical resection [11]. After the surgery, the patient experienced an improvement in visual acuity, and the visual field returned to normal within two months. At the time of writing, she had not experienced any relapses and was undergoing regular follow-up.

Postpartum regression of the tumor has been observed in some cases, presumably due to the normalization of hormone levels after pregnancy. However, certain factors, such as higher-grade tumors, larger tumor size, and certain histological subtypes, may influence long-term outcomes. Thus, long-term monitoring is essential as there is always a risk of recurrence.

## 4 Conclusion

Meningiomas during pregnancy present unique challenges and can have a wide array of clinical presentations. Pregnancy is considered risky and patients should be carefully monitored for possible neurological maternal manifestations and fetal well-being. Timely diagnosis, personalized treatment strategies, and close monitoring are essential for achieving favorable outcomes and the timing of delivery should be based on an accurate evaluation of the fetal and maternal outcomes. The presence of a multidisciplinary team is essential to improve maternal and fetal survival. Further research is needed to enhance the understanding of the underlying mechanisms and improve management approaches for this rare condition.
